# Supplementary material for: Crystal Structures of Inhibitor-Bound Main Protease from Delta- and Gamma-Coronaviruses
Source: Viruses. 2023 Mar 18;15(3):781. doi: 10.3390/v15030781 (PMC10059799; doi:10.3390/v15030781)
Supplement: Supplementary file 1 [file viruses-15-00781-s001.zip › viruses-2223519-supplementary.pdf]

SUPPLEMENTARY MATERIALS

Crystal Structures of Inhibitor-Bound Main Protease from Delta- and Gamma-Coronaviruses

Sarah N. Zvornicanin[1], Ala M. Shaqra[1], Qiu Yu J. Huang[1], Elizabeth Ornelas[2], Mallika Moghe[3], Mark Knapp[2], Stephanie Moquin[2], Dustin Dovala[2], Celia A. Schiffer[1] and Nese Kurt Yilmaz[1]

[1] Department of Biochemistry and Molecular Biotechnology, University of Massachusetts Chan Medical School, Worcester, Massachusetts 01605, USA
[2] Novartis Institutes for Biomedical Research, Emeryville, CA 94608, USA
[3] Department of Molecular and Cell Biology, University of California, Berkeley, CA 94720, USA
* Correspondence: Nese.KurtYilmaz@umassmed.edu

Table S1: Crystallization and refinement statistics of Mpro structures for SARS-CoV-2 (SC2), beluga whale (SW1) and swine (HKU15) coronaviruses.

M^{pro}–Inhibitor	***SC2–PF-00835231***	***SW1 apo***	***SW1–GC376***	***HKU15–PF-00835231***
PDB ID	8DSU	8FWX	8E7N	8E7C
Data Collection				
Location	Home source	ALS 5.0.2	Home source	Home source
Resolution range (Å)	27.11-1.86 (1.926-1.86)	68.03 – 2.11 (2.193 – 2.11)	12.24-1.65 (1.709-1.65)	22.91-2.45 (2.538-2.45)
Space group	$P2_1$	$P2_12_12$	$P2_1$	$P6_1$
a, b, c (Å)	54.9, 98.9, 59.1	81.8, 136.0, 49.9	63.1, 84.2, 68.5	64.1, 64.1, 261.8
α, β, γ (º)	90, 107.6, 90	90, 90, 90	90, 93.1, 90	90, 90, 120
Total reflections	242993 (15914)	207173 (26479)	199032 (15277)	50645 (4137)
Unique reflections	50477 (5011)	32590 (4634)	81531 (7698)	20849 (1863)
Multiplicity	4.8 (3.2)	6.4 (5.7)	2.4 (2.0)	2.4 (2.2)
Completeness (%)	99.90 (99.60)	99.9 (99.2)	94.95 (90.26)	93.51 (83.92)
Average I/σ	19.97 (1.85)	10.7 (1.7)	11.31 (1.61)	5.37 (1.26)
Wilson B-factor	23.66	38.46	13.90	24.10
R_{merge}^{a}	0.04739 (0.6403)	0.091 (0.867)	0.05568 (0.4862)	0.1608 (0.6731)
$CC_{1/2}$	0.999 (0.738)	0.998 (0.663)	0.997 (0.735)	0.973 (0.452)
Refinement				
R_{factor}^{c}	0.2049 (0.3112)	0.1988 (0.2933)	0.1593 (0.2368)	0.1927 (0.2385)
R_{free}^{d}	0.2543 (0.3744)	0.2379 (0.3389)	0.1951 (0.2747)	0.2349 (0.2937)
$RMSD^{b}$ in:				
Bond lengths (Å)	0.004	0.003	0.018	0.002
Bond angles (º)	0.55	0.61	1.50	0.48
Ramachandran:				
Favored (%)	97.35	98.17	98.48	94.19
Allowed (%)	2.65	1.67	1.52	5.13
Outliers (%)	0	0.17	0	0.68
Rotamer outliers (%)	0.99	0.64	0	3.85
B-factors:				
Average	27.81	49.17	19.98	25.30
Macromolecules	26.54	50.09	17.56	24.98
Solvent	37.46	45.80	33.40	26.05

[a]Rsym = Σ | I − <I>|/ Σ I, where I = observed intensity, <I> = average intensity over symmetry equivalent.

[b]RMSD, root mean square deviation.

[c]Rfactor = Σ || Fo| − |Fc||/ Σ|Fo|.

[d]Rfree was calculated from 5% of reflections, chosen randomly, which were omitted from the refinement process. Statistics for the highest-resolution shell are shown in parentheses.

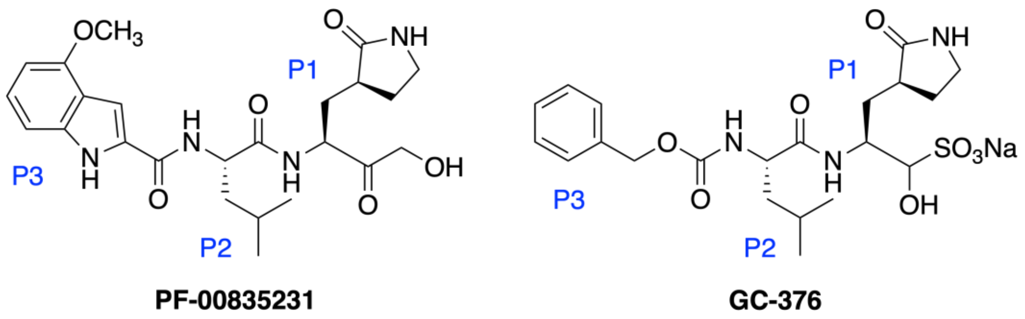

Figure S1. Structures of the M^pro^ inhibitors in the determined cocrystal structures, with the P1–P3 moieties labeled.

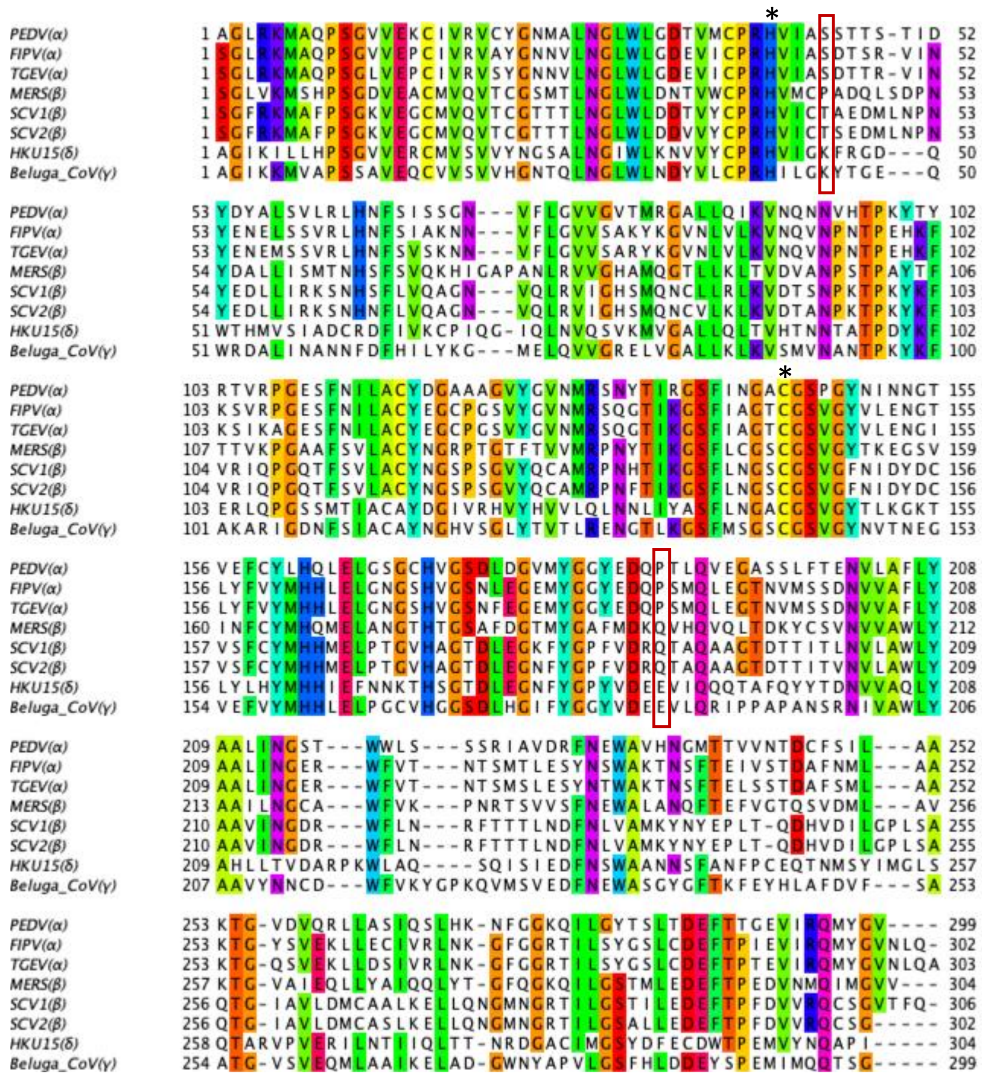

Figure S2. Amino acid sequence alignment of M^pro^ from various coronavirus species. The conserved catalytic dyad residues (His and Cys) are indicated by asterisk, and the amino acids forming the salt bridge over the S2 subsite in delta- and gamma-CoV are highlighted in red frame. Alpha-CoVs are PEDV (porcine endemic diarrhea virus), FIPV (feline infectious peritonitis virus), TGEV (transmissible gastroenteritis virus); beta-CoV are MERS (Middle East respiratory syndrome), SCV1 (SARS-CoV-1), SCV2 (SARS-CoV-2), delta-CoV is the porcine HKU15 and the gamma-CoV is the beluga whale SW1. Multiple sequence alignment was performed using the Clustal web service with default parameters through the Jalview menu. The sequences are colored using the "Taylor" scheme with the threshold set at 60% identity.

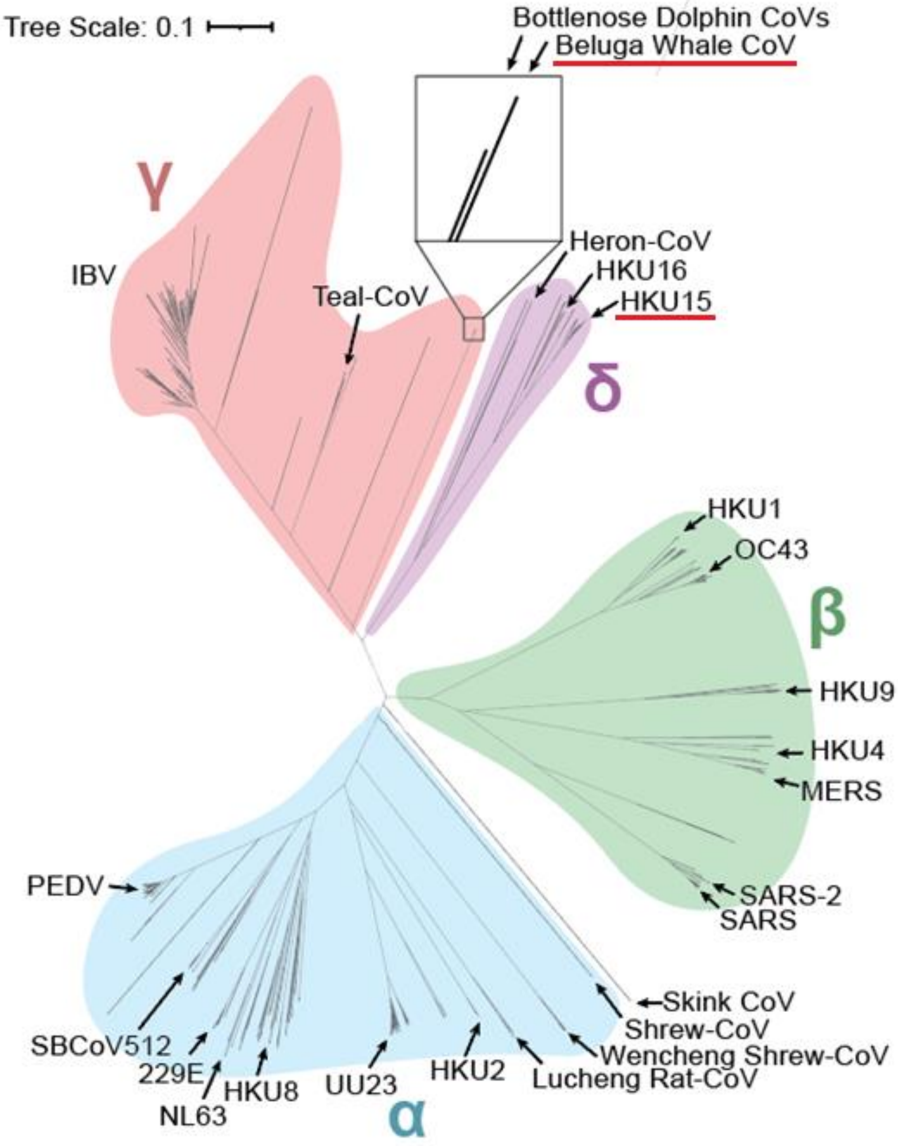

Figure S3. Phylogenetic tree of coronaviruses displaying the four main genera: alpha, beta, gamma, and delta. The tree was generated based on M^pro^ amino acid sequences. The beluga whale (SW1) and porcine (HKU15) coronaviruses of which M^pro^ structures were solved here are underlined.
